# Blood filtering system for COVID-19 management: novel modality of the cytokine storm therapeutics

**DOI:** 10.3389/fimmu.2023.1064459

**Published:** 2023-04-21

**Authors:** Vivek P. Chavda, Nidhi Raval, Soham Sheta, Lalitkumar K. Vora, Fatma Elrashdy, Elrashdy M. Redwan, Vladimir N. Uversky, Yavuz Nuri Ertas

**Affiliations:** ^1^ Department of Pharmaceutic and Pharmaceutical Technology, L M College of Pharmacy, Ahmedabad, India; ^2^ National Institute of Pharmaceutical Education and Research (NIPER) – Ahmedabad, Gandhinagar, Gujarat, India; ^3^ Formulation and Development, Zydus Lifesciences Ltd., Ahmedabad, Gujrat, India; ^4^ School of Pharmacy, Queen’s University Belfast, Belfast, United Kingdom; ^5^ Department of Endemic Medicine and Hepatogastroenterology, Cairo University, Cairo, Egypt; ^6^ Biological Science Department, Faculty of Science, King Abdulaziz University, Jeddah, Saudi Arabia; ^7^ Department of Molecular Medicine and Byrd Alzheimer’s Research Institure, Morsani College of Medicine, University of South Florida, Tampa, FL, United States; ^8^ ERNAM - Nanotechnology Research and Application Center, Erciyes University, Kayseri, Türkiye; ^9^ Department of Biomedical Engineering, Erciyes University, Kayseri, Türkiye

**Keywords:** cytokine storm, cytokine, blood filter, COVI-19, COVI-19 management, immune dysfunction

## Abstract

The newly emerged coronavirus (SARS-CoV-2) is virulent, contagious, and has rapidly gained many mutations, which makes it highly infectious and swiftly transmissible around the world. SARS-CoV-2 infects people of all ages and targets all body organs and their cellular compartments, starting from the respiratory system, where it shows many deleterious effects, to other tissues and organs. Systemic infection can lead to severe cases that require intensive intervention. Multiple approaches were elaborated, approved, and successfully used in the intervention of the SARS-CoV-2 infection. These approaches range from the utilization of single and/or mixed medications to specialized supportive devices. For critically ill COVID-19 patients with acute respiratory distress syndrome, both extracorporeal membrane oxygenation (ECMO) and hemadsorption are utilized in combination or individually to support and release the etiological factors responsible for the “cytokine storm” underlying this condition. The current report discusses hemadsorption devices that can be used as part of supportive treatment for the COVID-19-associated cytokine storm.

## 1 Introduction

The International Committee on Virus Taxonomy designated a novel virus Severe Acute Respiratory Syndrome Coronavirus 2 (SARS-COV-2) in February 2020 (1, 2). According to data from the World Health Organization (WHO), there have been nearly 583 million confirmed cases of COVID-19, and 64.1 million deaths have been reported. Viruses, including SARS-CoV-2, are constantly mutating, and new variants that are more dangerous than the original virus may emerge. These variants are classified by the WHO based on their risk potential (3). Mutations and genetic recombinations lead to the emergence of new SARS-CoV-2 variants that are more transmissible and have high immune evasion characteristics (4–7). These new emerging variants further complicate the COVID-19 management (8). Recently, a new viral outbreak, monkey pox, has also been impacting different parts of the globe (9).

The occurrence of the so-called “cytokine storm (CS)” has been linked to COVID-19 sufferers’ death (10). Abnormal production of proinflammatory cytokines exacerbates acute respiratory distress syndrome (ARDS) and destroys a great deal of tissue, which may result in organ failure and death (10). Systemic inflammatory syndromes like CS and cytokine release syndrome can be fatal. They are distinguished by high levels of circulating cytokines and overactive immune cells, which can be caused by a number of therapies, pathogens, cancers, autoimmune diseases, and monogenic disorders (11–14). Although new and repurposed drugs are typically considered as pharmacologic interventions for the treatment of COVID-19, the utilization of the extracorporeal circuits as a means to decrease the concentration and control the circulating levels of the inflammatory mediators represents an important and promising approach (15–17). The idea of pathogenic immune activation and its therapeutic corollary, immunosuppression for infection therapy, is both troubling and transformative. As a result, understanding the start and signaling mechanisms of CS is therapeutically crucial in order to design more effective COVID-19 therapy options. We review the most recent discoveries in the immunopathological features of COVID-19, with an emphasis on CS, and the current research status of the many cytokines implicated. We have majorly focused on the cytokine storm blood filtering systems for COVID-19 management; the same can also be used for the efficient management of CS associated with any fatal systemic inflammatory syndromes.

## 2 Cytokine storm - fuelling deaths in COVID-19 patients

**Figure 1:**
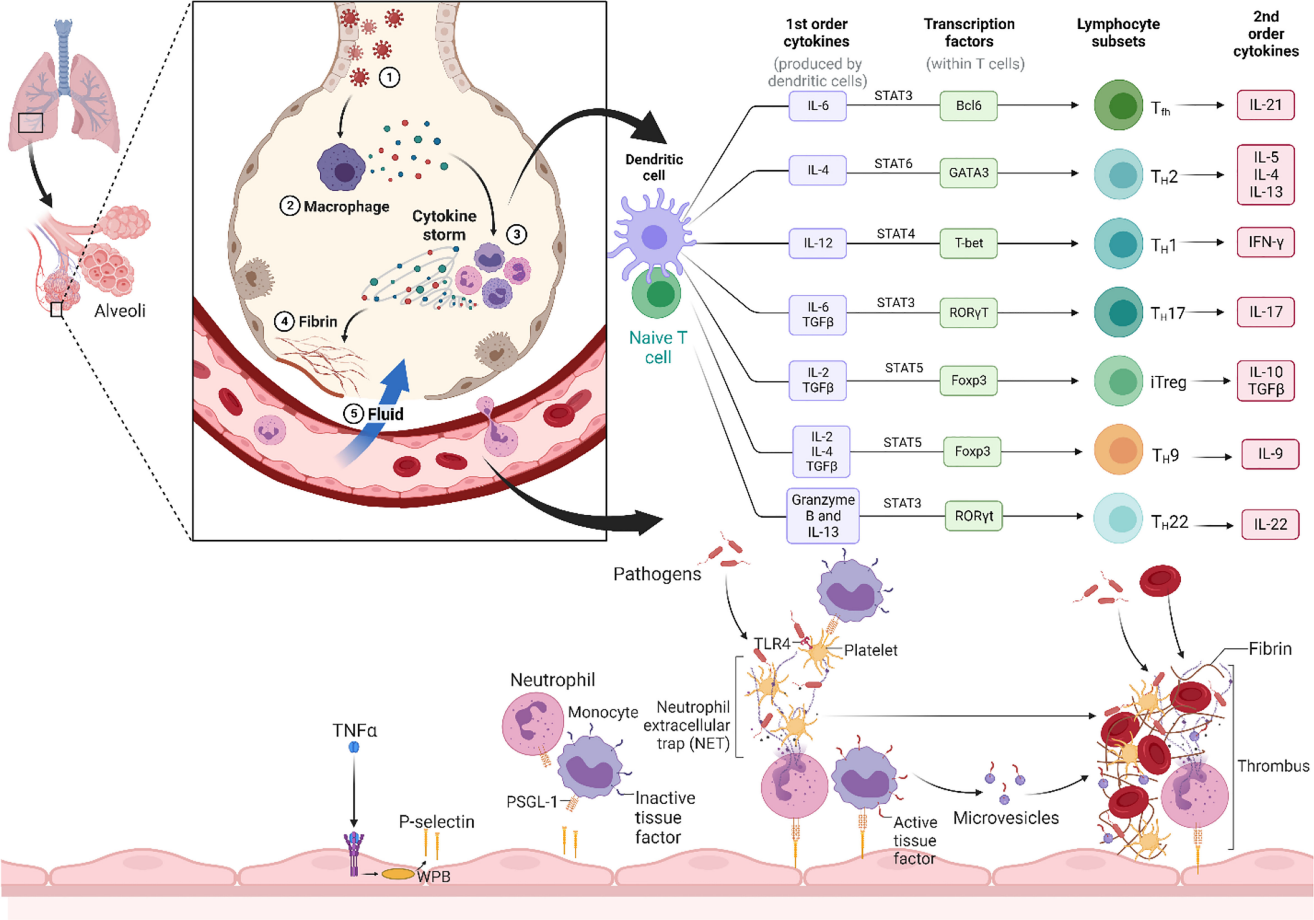
Overall mechanism of the cytokine storm. The cytokine storm is caused by SARS-CoV-2, which causes severe cytokine/chemokine reactions (Release of first order and second order cytokine like IL-1, IL-2, IL-4, IL-6, IL-7, IL-10, IL-12, IL-13, IL-17, M-CSF, G-CSF, GM-CSF, IP-10, IFN-γ, MCP-1, MIP 1-α, hepatocyte growth factor (HGF), TNF-α, and vascular endothelial growth factor (VEGF)) in certain people with the disease. Cytokine storm induces ARDS, also known as multiple-organ impairment that also leads to physiological deterioration and fatality. (Created with Biorender.com).

CS refers to the over releasing of cytokines into the blood quickly due to infection and other stimulants. Generally, understanding its pathophysiology is complicated, and the lack of regulatory control over pro-inflammatory cytokine production in local and systemic factors is ambiguous (18, 19). Some of the infected individuals witnessed considerable deterioration through the coronavirus disease (COVID-19), linked with cytokine dysregulation and overproduction (**Figure 1**). In response to SARS-CoV-2 infection, macrophages and dendritic cells initiate an early immunological response characterized by lymphocytosis and the production of cytokines. However, the inflammatory reaction leads to the elimination of lymphocytes that are attempting to combat SARS-CoV-2 infection. Lymphopenia develops, particularly in patients requiring ICU hospitalization due to the severity of their condition. Rapid dysregulation of cytokine production results in the destruction of healthy cells, primarily in the lungs but possibly also in the kidneys, heart, blood vessels, and brain. The cascade of damage caused by a cytokine storm starts with a breach in the lung’s epithelial barrier. The weakening of the epithelial barrier makes the lungs and other tissues susceptible to bacterial infection (20). Below, we discuss the physiopathology and treatment methods of the COVID-19 virus-induced inflammatory storm and illustrate some context for relevant treatment guidance.

### 2.1 Cascade of events leading to the immune dysregulation

The mechanism of the physiopathology of human coronaviruses (H-CoVs) such as SARS-CoV, Middle East respiratory syndrome–related coronavirus (MERS-CoV), and SARS-CoV-2 is rarely apparent, and they have all been linked to a disordered and overactive immune system response, especially in cytokine production (21). Cytokines are glycoproteins released by different types of cells that regulate a wide range of biological processes in an active interplay of innate and adaptive immune responses and play a vital role in preventing and treating infections and chronic illnesses (22). During the SARS-CoV-2 infection, cytokines play a significant role in regulating and resolving coronavirus infections. Uncontrolled, exaggerated production of numerous cytokines could cause and/or participate in immunopathogenesis by triggering catastrophic outputs throughout the body. This not only frequently exacerbates collateral tissue damage but also prolongs the course of the disease and causes an exponential increase in mortality rates. This urges the medical and biomedical scientific communities to find proper medications to control and manage infection, which should lead to a reduction in deaths, specifically in the severely ill COVID-19 patients (21).

In many cases, COVID-19 patients may experience moderate symptoms such as fever, cough, and even a missing taste or smell. However, some infected people may encounter cytokine release syndrome (CRS) as a critical and adverse complication. CRS is a crucial factor for COVID-19 individuals with severe symptoms. Several studies have recommended that hyper inflammation be identified and treated to decrease the occurrence of death and accelerate recovery. CRS occurs either directly due to viral damage or indirectly as a result of an overactive immune system that causes immune cells like macrophages and neutrophils to infiltrate the tissue (23). This reaction is a defensive response to prevent virus propagation under the conditions of tightly controlled levels of the cytokines. Unfortunately, in some cases, when this defensive response goes out of control, it has the opposite effect (24), which is further controlled by other circumstances, such as comorbidities and various genetic and environmental factors (25).

ARDS appears to be a common clinical outcome not limited to the three coronavirus infections (SARS-CoV-2, MERS-CoV, and SARS-CoV), but being observed in many other infectious and noninfectious diseases. The respiratory failure caused by ARDS has been the leading cause of death in these infections (26). ARDS is a condition of acute respiratory failure defined by increasing arterial hypoxemia, dyspnea, and a substantial increase in breathing in infected individuals with lung problems. Endotracheal intubation and positive pressure ventilation are required for the majority of severely ill patients. Notably, many ARDS patients suffer from extra-pulmonary organ failures, such as cardiovascular failure needing vasopressor support, renal failure requiring dialysis, abnormal liver function, anemia, and thrombocytopenia (27).

CS is a particular term that indicates the intensity and potential power of super-inflammatory conditions to cause tissue damage mediated by the patient’s immune response. Cytokine storm syndromes (CSS) are a group of disorders that share a clinical pattern of hyperferritinemia, hyperinflammation, and multiorgan failure. They are caused by the overproduction of cytokines, which is typically self-perpetuating due to unrestricted and enhanced immune activation (28). In addition to viral, oncologic, genetic, rheumatic, and iatrogenic etiologies, the range of cytokine storm disorders also includes rheumatic and iatrogenic diseases. The CSS is a valuable term to provide a pattern of shared clinical considerations and immune-mediated pathophysiology components. The CSS can be beneficial in recognizing hyperinflammatory conditions, understanding the drivers of the inflammation, and determining therapeutic options (29). With initial cases demonstrating remarkable clinical improvements in individuals treated with the anti-interleukin (IL-6) biologic tocilizumab (the standard treatment for CRS), the idea of a CSS in severe COVID-19 became more visible (30, 31).

As COVID-19 spreads around the world, a series of uncontrolled cases of anti-cytokines (mostly anti-IL-6 and anti-IL-1) are promising and show clear benefits in large-scale controlled trials with corticosteroids. COVID-19 has shown a considerable increase in the levels of several cytokines and chemokines in clinical tests (32, 33). The cytokine storm that occurs after sepsis is a critical factor in the development of ARDS, which is characterized by high levels of proinflammatory cytokines like interferon (IFN)-α, IFN-γ, IL-1β, IL-33, IL-18, IL-12, IL-6, tumor necrosis factor (TNF)-α, and chemokines such as CCL2, CCL3, CCL5, CXCL8, CXCL9, and CXCL10. In SARS-CoV infections, they are also released by immunological cells. Patients with severe MERS-CoV disease had higher serum levels of IL-6, IFN-α, and CCL5, CXCL8, and CXCL-10 than those with mild to moderate infection (34, 35). The massive cytokine storm responses in SARS-CoV-2 infection, as in SARS-CoV and MERS-CoV infections, exert a massive attack on the body due to excessive immune activation, causing ARDS and multiorgan failure and eventually death in severely affected individuals (35).

Immune function declines with immunosenescence in elderly people and illustrates a high rate of mortality (36). These phenomena occur when the body experiences a decrease in the generation of CD3+ T cells, an inverted CD4 to CD8 (CD4/CD8) T cell ratio owing to the loss of CD8+ T cells (increased CD4/CD8 ratio), or an increase in regulatory T cells and a decrease in B lymphocytes (37–39). In general, it is hypothesized that immunosenescence causes an induced cytokine storm in COVID-19. The virus can infect T lymphocytes, which induce apoptosis. In addition, when viruses and bacteria induce an inflammatory response locally known as hypercytokinemia, acute-response cytokines such as TNF and IL-1β and chemotactic cytokines such as IL-6 and MCP-1 elevate and lead to damage to organ systems. These regulations of IL-6 and MCP-1 activate the Janus kinase signal transducer and activator of transcription (JAK-STAT) pathway, and as a result, the inflammatory process continues (40). However, a study claimed that CCL5 levels in the peripheral blood of severe COVID-19 patients were greater than IL-6 levels (41). Therefore, blocking the stream level of cytokine response processes, such as macrophage JAK-STAT signaling to decline IL-1 and IL-6 production, might be a viable treatment option for cytokine storm.

In another study, the amplified progression of COVID-19 to the cytokine storm is classified as time-dependent in three phases. As a first stage known as the initiation phase, which occurs in 0-4 days, the immune system releases chemokines such as CCL2, CCL3, CCL5, IP-10, and cytokine IL-1A. In the second stage (amplification phase), which happens in 5-14 days, IL-2, IL-6, IL-10, CCL2, and CCL3 as inflammatory mediators are activated (42). Finally, in the third stage (consumption phase), other cytokine storms can lead to death after 15 days. These three phases progress quickly, with a median duration from beginning to death of 16–18.5 days, and comorbidities like cardiovascular illness can further accelerate the progression (43, 44).

As a simple definition of the mechanism for COVID-19, when high levels of cytokines and chemokines are induced in the region of the respiratory cells, the blood coagulation process and severe disease pathology are accomplished, which means that immune cells and their inflammatory mediators may contribute to thrombotic risk due to the electrostatic interactions between the neutrophil extracellular traps (NET) histones and platelet phospholipids in the capillary beds and vein (44–46).

### 2.2 Multi-organ involvement

COVID-19 is no longer merely a pulmonary epithelial infection but a multi-system inflammatory illness that leads to end-organ failure (47), through cross-talk *via* soluble and particulate multifactorial agents. There are antiviral medicines on the market, such as Remdesivir, which are useful in certain circumstances and are superior to placebo in reducing the time to recovery in hospitalized individuals with COVID-19 and evidence of a lower respiratory tract infection, but they are not a definitive and appropriate treatment. Therefore, the focus should be given to alternative methods of reducing end-organ degeneration while perfecting pharmaceutical options. According to reports and studies in hospitalized patients, multiorgan ischemia begins with a high secretion of cytokines and culminates in the cardio-renal syndrome, which loses its integrity due to hypoperfusion of blood vessels (48, 49).

Cardioprotection is the process of reducing damage to the myocytes after a myocardial infarction and stopping the problem of reperfusion (50–52). Because the heart is vital for circulatory function and multiorgan perfusion, cardiac protection in COVID-19 may have a multi-system benefit. There is a considerable immunological response after ST-elevation myocardial infarction, which causes systemic inflammation. Similarly, following plaque rupture, proinflammatory cytokines, inflammasomes, and immune-mediated thrombosis are upregulated (53). In cardioprotective research, identifying the Reperfusion Injury Salvage Kinase (RISK) pathway is a key achievement (54). When it is activated early in ischemia, RISK identifies a collection of pro-survival protein kinases that operate to limit cell death by lowering mitochondrial transition pore opening (51). When a “multi-hit” method is used to protect organs against tissue hypoxia, the results are satisfying (55). Numerous mechanisms can be targeted in cardioprotective pharmacology to prevent cardiac cell death and dangerous inflammation (55, 56). RISK, Survivor Activating Factor Enhancement (SAFE), and Nitrous Oxide/Protein Kinase (NO/PKG) are examples of pro-survival pathways. Significantly, these pathways in COVID-19 patients allow for the most effective application of this treatment at the outset of moderate disease, before the cytokine storm develops (57).

### 2.3 Current management practice

COVID-19 can be treated with a number of approved medications that have been shown to work and are safe. Common knowledge or acceptance-based management techniques suggest that most of the care for COVID-19 patients should be supportive. Therapeutic methods that target the cytokine storm in the pathogenesis of severe COVID-19 pneumonia need special investigation. According to current World Health Organization (WHO) recommendations, supportive care, including a decline in fever, fatigue, and relieving pain, is current management practice. In moderate cases, patients require oxygen therapy, intravenous fluids, and antihypertensive drugs to raise blood pressure and optimize supportive care treatments (58). For moderate to severe cases, immunosuppressive drugs sometimes provide essential corticosteroids and empiric antibiotic administration as needed, which remains the most critical management strategy for pneumonia (36).

Corticosteroids are synthetic counterparts of the endogenous steroid hormones of the adrenal cortex. These synthetic substances exhibit glucocorticoid and mineralocorticoid characteristics (59). Since steroid hormones are essential in reducing inflammation in the human body, corticosteroids are a group of therapeutic hormones that are used to support the reduction of inflammation (60). It is worth noting that the use of glucocorticoids is controversial. They are also used in both the adrenal (physiological) and super physiological doses to replace many dermatological, ophthalmological, rheumatological, pulmonary, hematological, and gastrointestinal illnesses. Doctors at the forefront of the fight against coronavirus in China are less inclined to use steroid drugs. However, the use of steroid drugs in low doses is lifesaving in some patients (61). Immunomodulatory clinical practitioners extensively employed corticosteroids during the SARS pandemic (2002–2003), and based on the clinical feedback, they could lead to early favorable changes, such as drop-in pyrexia, resolution of radiographic lung infiltration, and oxygenation improvement (62, 63). Studies demonstrate that opposition to the corticosteroid therapy method is rising since it has no advantage for patient survival and may cause harm in infected patients with coronavirus and influenza illnesses. Corticosteroids are not advised for individuals with human coronavirus infections in general, though they may be used cautiously in critically ill patients (64).

### 2.4 Recent updates for management of cytokine storm

Besides the blood purification system, an artificial liver blood purification system was also utilized to eliminate or reduce the cytokine storm in COVID-19 patients (65). They compared the outcomes of the pre-artificial liver-blood purification system to the results of the post-artificial liver-blood purification system. The outcome suggested that an artificial liver-blood purification system can stop cytokine storms and instantly eradicate inflammatory mediators, as well as reduce the progress of COVID-19 disease. Thus, it can be suggested as a new potential treatment modality to govern COVID-19. There is currently no established therapy for the COVID-19 viral infection, which infects the lower airway directly and causes widespread infection by activating multiple cytokine mediators, resulting in acute respiratory distress syndrome. A clinical trial (pilot study) was started on April 17, 2020, on 40 COVID-19 patients suffering from ARDS. The study was designed to assess the safety as well as the efficacy of the cytokine adsorption device and cytokine filtration. The results suggested that cytokine filtration is an effective treatment, and further clinical trials are also emphasized for safety assessment (66). Meanwhile, in 2021, Avalon Inc. planned the first human clinical trial of the AVA-Trap blood filtration system to eradicate cytokine storms. AVA-Trap is comprised of two technologies: QTY code and S-layer. AVA-Trap technology utilizes cytokine receptor Fc-fusion proteins and antibody-like traps that attract cytokines released by the immune system. The Fc-fusion proteins act as a molecular sponge, soaking up unwarranted cytokines. When the patient’s blood is circulated through the AVA-Trap device, the cytokine receptor trap attaches an S-layer matrix to trap proteins or excessive cytokines. AVA-Trap, as an innovative diagnostic tool, has the unique property to trap selective molecules from the blood and separate cytokines from other types of available molecules in COVID-19 patients (67). In addition, S-layer technology is simultaneously used by Avalon Inc. as an intranasal spray vaccine for COVID-19. It can be provided as first-line protection as soon as the virus enters the body. The S-layer protein-based intranasal vaccine is under phase 3 clinical trial and has proven to decrease SARS-CoV-2 infection severity and also can reduce respiratory inflammation and organ damage. It is found to be an effective and advantageous technology for the protection of the immune system in COVID-19 patients. Avalon Inc. started clinical trials in the US and China in Q4 of 2020 and Q1 of 2021 with CB-MSC-1 (allogeneic mesenchymal stromal cells-derived from human cord blood) (68). CB-MSC-1 has exceptional anti-inflammatory and immunomodulatory actions, along with the capability to reduce T cell propagation and cytokine excretion and control the stability of antibody-based and cell-based immune reactions. CB-MSC-1 has been proven safe and effective in the pre-clinical study, and recently, Avalon Inc. declared its intention to initiate a human clinical study for COVID-19 patients with acute respiratory distress syndrome (67).

## 3 ‘Cytokine storm’ blood filtering system for COVID-19 management

A small glycoprotein system such as cytokinesis is generated by several types of cells throughout the body. The release of cytokines activates various functions such as cell proliferation, differentiation, paracrine, autocrine, or endocrine activity and also triggers immune and inflammatory functions (e.g., interferons, interleukins, chemokines, colony-stimulating factors (CSFs), and tumor necrosis factor (TNF) (69). “Cytokine storm” was designated as hypercytokinemia, which was elaborated in 1993 to represent graft-versus-host disease. Cytokine storm is also referred to as an enhanced response of the body’s immune system, which can be lethal due to infectious diseases. The word “cytokine” is derived from the Greek words “cyto” (cell) and “kinos” (movement) (42). When SARS-CoV-2 invades the lungs and activates the immune response, immune cells induce inflammation locally. A release of an extreme number of cytokines can activate more and more immune cells, which leads to hyperinflammation. This cell activation cascade can cause serious harm to the patient and even be fatal.

Cytokine storms are often a problem for COVID-19 and the flu, as well as for other lung diseases like SARS and MERS. Diseases that aren’t contagious, like multiple sclerosis and pancreatitis, can also cause these reactions (22). The symptom became more well recognized following the 2005 outbreak of the H5N1 influenza virus, often known as “bird flu,” when the mortality rate increased due to an out-of-control cytokine response (70, 71). These are major symptoms observed in COVID-19 patients and may describe the difference in symptom severity among patients. For example, younger people may show weaker symptoms due to having less developed immune systems and lower levels of cytokines (72).

In the course of the SARS epidemic (SARS-CoV), the term “cytokine storm” was also explained. Numerous early instances of COVID-19 revealed blood cytokine levels that exceeded the normal limits. IL-6 as a pro-inflammatory cytokine is a key facilitator for the acute inflammatory response as well as the suspected cytokine storm (73). At present, filtrating out of cytokines is another predicted approach to alleviating the devastating admission of patients to intensive care units. Furthermore, CytoSorb is a potentially suitable means to aid COVID-19 patients more efficiently and release them from the intensive care unit to improve the situation. Although CytoSorb is not able to eradicate the virus, it has been utilized in over 80,000 European practices as proper management for cytokine storms. It has been globally disseminated to more than 55 countries and helps doctors control the overpowering inflammation, gradually reverse the shocks, and increase breathing and other related functions of the heart, which are the main therapeutic measures of COVID-19 infection (74).

## 4 Blood filtration strategies for COVID-19 management

Many approaches have been elaborated to decrease the concentration and control the circulating levels of the inflammatory mediators. These mediators can be eliminated from plasma *via* the extracorporeal circuit, which can reduce the deleterious effects of such mediators. There are several hemadsorption extracorporeal methods that utilize CytoSorb cartilage and related high cut-off membranes. High volume fluid exchange reduces serum concentration while increasing lymphatic flow, resulting in a proportionate reduction of the concentration of the cytokines in the tissues (75). Since extracorporeal methods can reduce the concentration of certain cytokines in the blood, early blood filtering can potentially mitigate cytokine storms before irreversible damage occurs and thereby help to avoid clinical manifestations.

### 4.1 Cytoadsorbant^®^ (CytoSorb)

**Figure 2:**
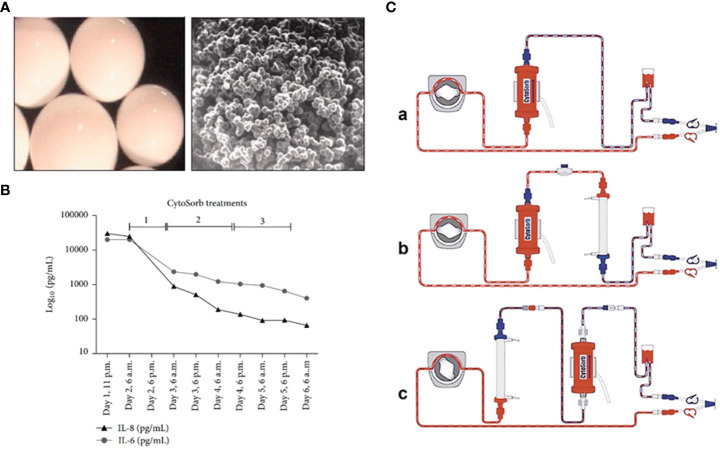
**(A)** Microscopic image for CytoSorb; **(B)** level of cytokines after incorporation of CytoSorb column; **(C)** formation of CytoSorb: **(a)** CytoSorb alone, **(b)** CytoSorb with pre dialyzer, **(c)** CytoSorb with post dialyzer. Reproduced with permission from (76–78).

CytoSorb, or cytosorbent, is a hemoadsorption column or complete blood filtration unit that comprises highly porous adsorbent polymer beads that are capable of eliminating inflammatory mediators from the blood **(** **Figures 2A–C**). The beads are composed of biocompatible polystyrene, divinylbenzene, and polyvinylpyrrolidone, which can adsorb numerous inflammatory mediators through size exclusion (< 55 kDa) as well as hydrophobic adsorption. The given filter provides a 45,000 m^2^ surface area for adsorption (79). The adsorption provided by this filter is non-selective and depends on concentration; therefore, at higher concentrations, substances are adsorbed at high rates. It is also able to eliminate low-molecular-weight substances such as albumin-coupled substances and pathogenic toxins, besides not being able to eradicate endotoxins. The report from the USFDA on April 15, 2020, approved emergency utilization of CytoSorbant as CytoSorb technology for COVID-19 patients, where the body’s hyper-vigilant immune system begins to attack its own cells (80). Numerous available pharmaceutical modalities are not completely able to eradicate the coronavirus. This blood filtration technology is capable of treating another type of immune system disease that can be a promising therapy for severe COVID-19 patients. COVID-19 is a life-threatening virus infection that overdrives the immune system, triggering inflammatory mediators like cytokines and producing even more cytokines (called a cytokine storm), further leading to worse viral pneumonia. The body’s inflammatory cascade also damages major organs such as the lungs, heart, and kidney (81). CytoSorb is a hemoadsorption column that is capable of eliminating inflammatory mediators from the blood. It comprises absorbent-coated beads **(** **Figures 2A–C** **)**. The polyvinylpyrrolidone-coated absorbent beads can improve biocompatibility. Meanwhile, the CytoSorb column is also able to be configured as well as added to the extracorporeal circuit, pre-dialyzer, or post-dialyzer. As a blood purifying cartridge, it can decrease cytokine storm intensity and complications by physical binding and eliminating cytokines from blood, much like dialysis (76).

Initially, septic shock patients developed multiple organ failure (MOF), which was associated with continuous veno-venous hemodiafiltration owing to acute kidney injury. At that time, the levels of IL-6 and IL-8 were dramatically raised beyond 5,000 pg/ml, and CytoSorb was inserted into the hemodiafiltration circuit. This filtration lasted for sixty hours, and the abundance of IL-6 and IL-8 decreased significantly **(** **Figure 2C** **)** (77). Secondly, the patient whose illness developed severely and who faced advanced respiratory damage during laparotomy was routinely kept on ventilation. Even after that, the condition of the patient worsened, and he also developed right ventricular failure; therefore, he had to switch to veno-arterial extracorporeal membrane oxygenation (ECMO). In addition, for patients with cardiopulmonary dysfunction who have failed traditional treatment, ECMO has proven to be a vital intervention. ECMO is often used in one of two modes: veno-venous (V-V) or veno-arterial (V-A). V-v ECMO is used in chronic lung impairment, with good outcomes in bridging to recovery and lung transplantation. Due to the rapid invasion of inflammatory mediators such as IL-6 and IL-8, the patient’s blood was filtered through the CytoSorb column with Continuous Renal Replacement Therapy (CRRT) and antibiotics. After 48 hours, the extent of IL-6 and IL-8 was reduced continuously, and eventually, the patient recovered (82).

CytoSorb, as a cytokine filter, is also issued in the European Union and utilized in over 80,000 conditions, such as sepsis, lung failure, extremely low blood pressure, or shock. More than 70 critically ill COVID-19 patients in Italy, China, Germany, and France have been treated with CytoSorb with positive results in terms of regulating the cytokine storm and improving lung function, which helps keep COVID-19 patients from requiring mechanical ventilation. The Italy Brescia Renal COVID Task Force made an official endorsement to explicitly utilize Cytosorb in severe COVID-19 patients with Stage 3 acute kidney injury (AKI) and patients getting CRRT (80, 83).

**Figure 3:**
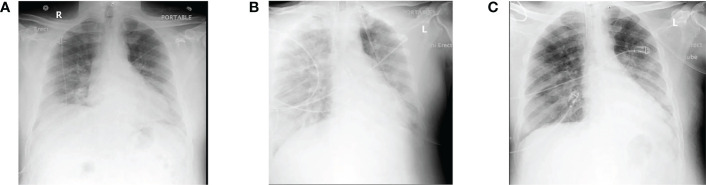
**(A)** Representation of the initial X-ray when the patient was admitted; **(B)** at day 6 of hospitalization representing severe and unremitting hypoxia and fever, with continued bilateral lung infiltration; **(C)** lung X-ray of cytokine filtration therapy at the first day demonstrating continuous infiltration with increased aeration with alleviated right lower lobe consolidation. Reproduced with permission from (80).

For instance, a patient suffering from COVID-19 (a 51-year-old male) with pulmonary inflammation and kidney malfunction was kept on CRRT, and the whole situation began to mimic a cytokine storm. Therefore, CytoSorb was applied to control the cytokine storm every 12 hours for the first 48 hours and then every 24 hours after that. Due to the damaged kidney condition, the patient received cytokine filter treatment along with CRRT. After three weeks, the IL-6 level was found to be stable, and hemodynamic stability was achieved. From this study, it can be concluded that CytoSorb is effective in reducing the severity of lung damage during a COVID-19 infection. Also, it was observed that early administration of blood filtration might significantly alleviate cytokine storms before possible irreversible damage occurs, hence allowing for a more effective therapy **(** **Figure 3** **)** (84).

The cytokine filter therapy has shown a clear possible benefit, but further research is required to prove the effect and make sure that this treatment method can be used in the future. The medical practice also believes that cytokine filter therapy leads to averting imminent death because it prevents the severe inflammatory response (cytokine storm) and prolongs the patient’s life. Cytokine filter therapy also supports critically ill patients and is also able to stabilize blood components with oxygen supplies as well as inflammatory markers (85). Consequently, a request has been submitted to the USFDA by CytoSorbents Inc. for a CytoSorb blood purification device to be utilized for COVID-19 in emergency cases. Clinical reports were able to reveal its efficacy *via* the effective elimination of inflammatory mediators, and based on those reports, it was authorized by the USFDA for critically ill COVID-19 patients (86).

### 4.2 Auxiliary membranes

**Figure 4:**
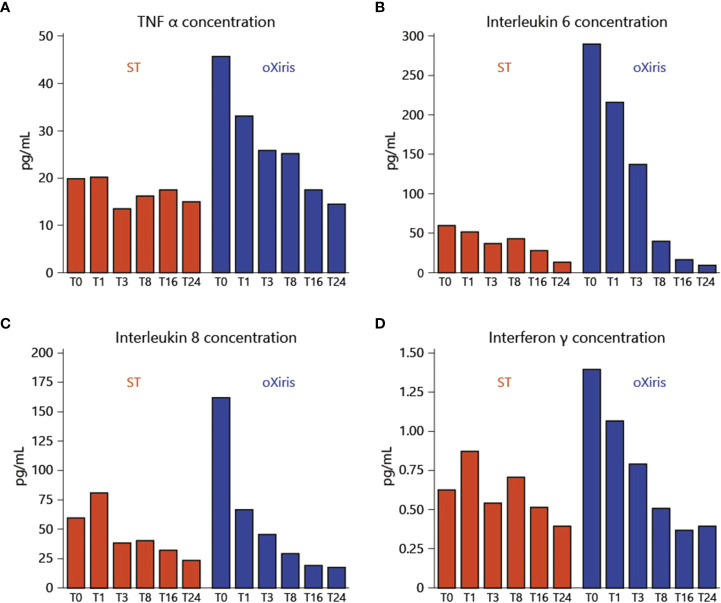
Inflammatory mediator levels before the beginning of treatment and throughout the filtration treatment *via* oXiris membrane: **(A)** TNF-α; **(B)** Interleukin-6 (IL-6); **(C)** Interleukin-8 (IL-8); **(D)** Interferon-γ. Reproduced with permission from (87).

The oXiris filter exhibits exceptional coating compared to the AN69 hemodialysis filter to obtain cytokine filtration and remove endotoxins, as well as anti-thrombogenic properties. In 1969, the first AN69 filter was made out of an acrylonitrile co-polymer and sodium methallyl. A negatively charged sulfonate group embedded in the co-polymer also had the ability to absorb cytokines. The filter interacting with the blood results in bradykinin generation, resulting in an anaphylactic reaction, particularly in patients receiving angiotensin converting enzyme (ACE) inhibitor therapy. Polyethyleneimine (PEI) was applied to the AN69’s exterior in order to eliminate this issue. The AN69-PEI coating may enhance biocompatibility by limiting bradykinin production and heparin adsorption, therefore permitting priming of the filter with heparin prior to use to enhance thrombogenicity. In the first 24 hours, patients receiving CRRT exhibited a substantial reduction in overexpressed cytokines (**Figure 4**). Molecular weight cutoff membranes (20-50 kDa) are also able to remove inflammatory mediators from COVID-19 patients within 24-48 hours (88). Another type of oXiris membrane (AN69), which is a cast of polyethyleneimine and embedded with heparin, is also utilized to reduce inflammatory cytokines in septic shock patients. In the first 24 hours, it showed a significant decrease of overexpressed cytokines in CRRT-receiving patients (**Figure 4**). Baxter Healthcare Corp. requested that the USFDA issue emergency authorization for the use of the oXiris Set device with AN69 membrane microstructure for the treatment of COVID-19 patients (over eighteen years of age) who were admitted to the intensive care units (ICUs) (under Section 564(d) (1)). It can be useful for COVID-19 patients suffering from severe respiratory failure who need blood purification with the intention of reducing inflammatory cytokines. The oXiris set device was found effective and was also able to remove cytokines from blood (89, 90)

In a swine model of acute kidney injury caused by lipopolysaccharide (LPS), CRRT with polysulfone membrane (PS-CVVH) was compared to CRRT with polymethylmethacrylate (PMMA) membrane (PMMA-CVVH) in terms of its ability to filter cytokines. Results suggested that PMMA-CVVH treatment significantly decreased LPS-binding protein levels in circulation compared to LPS levels, whereas there was no significant change observed between the PS-CVVH and LPS groups. Therefore, new PMMA with CVVH has a great impact on controlling systemic inflammatory syndrome as well as cytokine abundance (91). Another synthetic resin hemofilter (HA330) was used for cytokine regulation during cytokine storms. It was also investigated in acute pancreatic patients, and each cycle of filtration consisted of high-volume hemofiltration with hemoperfusion utilizing HA330. It was found that the combination approach for filtration was able to decrease inflammatory cytokines significantly (92).

ExThera Medical Corp. also requested USFDA approval for the emergency use of the Seraph 100 Microbind Affinity Blood Filter device and the extracorporeal blood purification (EBP) device for ICU-admitted COVID-19 patients to alleviate the cytokine storm. It was not completely approved initially *via* the USFDA, but based on the clinical data and its effectiveness as a blood filtration device to reduce numerous pathogens as well as inflammatory cytokines in COVID-19 patients, it was authorized to be utilized under Section 564(c) for emergency use only with suggested terms and conditions (87).

### 4.3 Coupled plasma filtration adsorption

Another type of extracorporeal filtration technique is coupled plasma filtration adsorption, utilized for plasma removal from blood, where the removed plasma passes through sorbent and then moves back into the blood. The sorbent was found to be non-specific for cells and also removed cytokines from the plasma. In a recent study where 35 patients with severe sepsis and acute kidney injury received coupled plasma filtration and adsorption with additional antimicrobials, after 10 hours of treatment for three days, a drastic reduction in IL-6, TNF-α, IL-8, and IL-10 was observed compared to the control patients (93). Overall, IL-6 is one of the substantial inflammatory factors involved in cytokine storms and mostly escalates vascular permeability and cardiac function. Therefore, recent studies and hospital practices mostly endorse the use of blood filtration devices along with current treatment modalities to decrease the number of ICU-admitted patients (94).

## 5 Concluding remarks

Although circulating SARS-CoV-2 infection has several dramatic economic impacts at the personal, community, country, and whole world levels, personal and population health remain the biggest concerns. It systematically tests our resilience at all these levels. The first six months of the COVID-19 pandemic resulted in 31.4 million confirmed SARS-CoV-2 infection cases and took over a million lives. We now have the means to reduce SARS-CoV-2 transmissibility and limit the COVID-19-related death rate. Among these measures are pharmaceutical interventions for controlling COVID-19 and managing its causative agent, as well as several COVID-19 vaccines. However, we continue to pay a high price, and several outstanding questions remain unanswered. One of these vital questions is how to control the cytokine storm induced by the SARS-CoV-2 infection. Although new and repurposed drugs are typically considered as pharmacologic interventions for the treatment of COVID-19, the utilization of the extracorporeal circuits as a means to decrease the concentration and control the circulating levels of the inflammatory mediators represents an important and promising approach. The corresponding devices can help eliminate the mediators from the plasma, thereby reducing their deleterious effects. In fact, extracorporeal hemadsorption devices (ECHAD), which are capable of filtering out the exacerbated mediators associated with COVID-19 pandemics, are included in general ICU clinical practice and undergo clinical trials. Although they had already demonstrated efficacy and safety, many questions were raised during their use in clinical practice and clinical trials. As the efficiency of these instruments depends on the filtration cut-offs, many COVID-19 medications used during the ECHAD-based procedures, as well as many important proteins, bioactive molecules, and even gases, are also filtered out. A large national cohort analysis of the 1,182 COVID-19 patients with ARDS who underwent ECMO therapy and the extracorporeal life support organization (ELSO) registry analysis of 1,032 patients who underwent ECMO for management of ARDS related to COVID-19, the mortality rates of such patients were found to be 45.9% and 39%, respectively (89). Although these numbers seem high, one should keep in mind that the patients undergoing ECMO therapy are those who have failed aggressive ventilatory support, thereby representing a sicker patient population when compared to the non-ECMO patients. The fact that ECMO therapy may save lives is further illustrated by the fact that the mortality rates for the 18–64-year-old COVID-19 patients with ARDS who were managed with and without ECMO therapy were comparable (44.6% vs. 37.9%, respectively) (90). Therefore, one cannot ignore the reasonable benefits of these technologies in the recovery of many severely ill COVID-19 patients (95). Taken together with the persistent challenge of the continuous COVID-19-associated loss of lives, the usefulness of the utilization of ECHAD alone or in combination with ECMO should be carefully evaluated in tightly controlled multicenter clinical trials.

## Author contributions

VC: Conceptualization, formal analysis, writing - original draft, writing-review & editing. NR and SS: writing - original draft. FE and EMR: writing-review & editing. LV, VU and YE: Supervision; writing-review & editing. All authors contributed to the article and approved the submitted version. **Figure 1** was created with BioRender.com.

## References

[1] Chavda VP Bezbaruah R Dolia S Shah N Verma S Savale S. Convalescent plasma (hyperimmune immunoglobulin) for COVID-19 management: An update. Process Biochem Barking Lond Engl (2023) 127:66–81. doi: 10.1016/j.procbio.2023.01.018 PMC9886570 36741339

[2] Ertas YN Mahmoodi M Shahabipour F Jahed V Diltemiz SE Tutar R. Role of biomaterials in the diagnosis, prevention, treatment, and study of corona virus disease 2019 (COVID-19). Emergent Mater (2021) 4:35–55. doi: 10.1007/s42247-021-00165-x 33748672 PMC7962632

[3] Chavda VP Bezbaruah R Valu D Patel B Kumar A Prasad S. Adenoviral vector-based vaccine platform for COVID-19: Current status. Vaccines (2023) 11(2):432. doi: 10.3390/vaccines11020432 36851309 PMC9965371

[4] Chavda VP Hanuma Kumar Ghali EN Yallapu MM Apostolopoulos V. Therapeutics to tackle omicron outbreak. Immunotherapy (2022) 8:833–8. doi: 10.2217/imt-2022-0064 PMC9180252 35678049

[5] Rashidzadeh H Danafar H Rahimi H Mozafari F Salehiabar M Rahmati MA. Nanotechnology against the novel coronavirus (severe acute respiratory syndrome coronavirus 2): diagnosis, treatment, therapy and future perspectives. Nanomed (2021) 16(6):497–516. doi: 10.2217/nnm-2020-0441 PMC7938776 33683164

[6] Chavda VP Apostolopoulos V. Is booster dose strategy sufficient for omicron variant of SARS-CoV-2? Vaccines (2022) 10:367. doi: 10.3390/vaccines10030367 35334999 PMC8950261

[7] Thakur P Thakur V Kumar P Singh Patel SK. Emergence of novel omicron hybrid variants: BA(x), XE, XD, XF more than just alphabets. Int J Surg (London England) (2022) 104:106727. doi: 10.1016/j.ijsu.2022.106727 PMC9225928 35753656

[8] Chavda VP Baviskar KP Vaghela DA Raut SS Bedse AP. Nasal sprays for treating COVID-19: a scientific note. Pharmacol Rep (2023) 27:249–65. doi: 10.1007/s43440-023-00463-7 PMC9969373 36848033

[9] Chavda VP Apostolopoulos V. Rare monkeypox: Is it really a threat to the elderly? Maturitas (2022) 15:90–1. doi: 10.1016/j.maturitas.2022.05.014 PMC9192136 35710608

[10] Ragab D Salah Eldin H Taeimah M Khattab R Salem R. The COVID-19 cytokine storm; what we know so far. Front Immunol (2020) 11:1446. doi: 10.3389/fimmu.2020.01446 32612617 PMC7308649

[11] Chavda VP Kapadia C Soni S Prajapati R Chauhan SC Yallapu MM. A global picture: therapeutic perspectives for COVID-19. Immunotherapy (2022) 14(5):351–71. doi: 10.2217/imt-2021-0168 PMC8884157 35187954

[12] Shimabukuro-Vornhagen A Gödel P Subklewe M Stemmler HJ Schlößer HA Schlaak M. Cytokine release syndrome. J Immunother Cancer (2018) 6:56. doi: 10.1186/s40425-018-0343-9 29907163 PMC6003181

[13] Zang S Chen Q Zhang Y Xu L Chen J. Comparison of the clinical effectiveness of AN69-oXiris versus AN69-ST filter in septic patients: A single-centre study. Blood Purif (2022) 51(7):617–29. doi: 10.1159/000519166 34610595

[14] Chavda VP Teli D Balar PC Vaghela D Solanki HK Vaishnav A. Potential anti-SARS-CoV-2 prodrugs activated by phosphorylation and their role in the aged population. Mol Basel Switz (2023) 28(5):2332. doi: 10.3390/molecules28052332 PMC10004871 36903575

[15] Chavda VP Hossain MK Beladiya J Apostolopoulos V. Nucleic acid vaccines for COVID-19: A paradigm shift in the vaccine development arena. Biologics (2021) 1(3):337–56. doi: 10.3390/biologics1030020

[16] Yamamoto N Bauer G. Apparent difference in fatalities between central Europe and East Asia due to SARS-COV-2 and COVID-19: Four hypotheses for possible explanation. Med Hypotheses (2020) 144:110160. doi: 10.1016/j.mehy.2020.110160 32795831 PMC7403102

[17] Kousi T Mitsi LC Simos J. The early stage of COVID-19 outbreak in Greece: A review of the national response and the socioeconomic impact. Int J Environ Res Public Health (2021) 18(1):322. doi: 10.3390/ijerph18010322 33406780 PMC7795843

[18] Pandiar D Kumar NS Anand R Kamboj M Narwal A Shameena PM. Does COVID 19 generate a milieu for propagation of mucormycosis? Med Hypotheses (2021) 152:110613. doi: 10.1016/j.mehy.2021.110613 34087613 PMC8152198

[19] Sarfraz Z Sarfraz A Jaiswal V Poudel S Bano S Hanif M. The past, present and future of COVID-19 associated mucormycosis: A rapid review. J Prim Care Community Health (2022) 13:21501319221099476. doi: 10.1177/21501319221099476 35587142 PMC9127848

[20] Bhaskar S Sinha A Banach M Mittoo S Weissert R Kass JS. Cytokine storm in COVID-19–immunopathological mechanisms, clinical considerations, and therapeutic approaches: The REPROGRAM consortium position paper. Front Immunol (2020) 11:1648. doi: 10.3389/fimmu.2020.01648 32754159 PMC7365905

[21] Harrison AG Lin T Wang P. Mechanisms of SARS-CoV-2 transmission and pathogenesis. Trends Immunol (2020) 41(12):1100–15. doi: 10.1016/j.it.2020.10.004 PMC7556779 33132005

[22] Bartee E McFadden G. Cytokine synergy: an underappreciated contributor to innate anti-viral immunity. Cytokine (2013) 63(3):237–40. doi: 10.1016/j.cyto.2013.04.036 PMC3748162 23693158

[23] dos Santos WG. Natural history of COVID-19 and current knowledge on treatment therapeutic options. BioMed Pharmacother (2020) 129:110493. doi: 10.1016/j.biopha.2020.110493 32768971 PMC7332915

[24] Moore JB June CH. Cytokine release syndrome in severe COVID-19. Science (2020) 368(6490):473–4. doi: 10.1126/science.abb8925 32303591

[25] Elrashdy F Redwan EM Uversky VN. Why COVID-19 transmission is more efficient and aggressive than viral transmission in previous coronavirus epidemics? Biomolecules (2020) 10(9):1312. doi: 10.3390/biom10091312 32933047 PMC7565143

[26] Xu Z Shi L Wang Y Zhang J Huang L Zhang C. Pathological findings of COVID-19 associated with acute respiratory distress syndrome. Lancet Respir Med (2020) 8(4):420–2. doi: 10.1016/S2213-2600(20)30076-X PMC7164771 32085846

[27] Matthay MA Zemans RL. The acute respiratory distress syndrome: pathogenesis and treatment. Annu Rev Pathol (2011) 6:147–63. doi: 10.1146/annurev-pathol-011110-130158 PMC3108259 20936936

[28] Ombrello MJ Schulert GS. COVID-19 and cytokine storm syndrome: are there lessons from macrophage activation syndrome? Transl Res J Lab Clin Med (2021) 232:1–12. doi: 10.1016/j.trsl.2021.03.002 PMC7934701 33684592

[29] Horby PW Mafham M Bell JL Linsell L Staplin N Emberson J. Lopinavir–ritonavir in patients admitted to hospital with COVID-19 (RECOVERY): a randomised, controlled, open-label, platform trial. Lancet (2020) 396(10259):1345–52. doi: 10.1016/S0140-6736(20)32013-4 PMC7535623 33031764

[30] Angus DC Derde L Al-Beidh F Annane D Arabi Y Beane A. Effect of hydrocortisone on mortality and organ support in patients with severe COVID-19: The REMAP-CAP COVID-19 corticosteroid domain randomized clinical trial. JAMA (2020) 324(13):1317–29. doi: 10.1001/jama.2020.17022 PMC7489418 32876697

[31] Williams AE Chambers RC. The mercurial nature of neutrophils: still an enigma in ARDS? Am J Physiol - Lung Cell Mol Physiol (2014) 306(3):L217–30. doi: 10.1152/ajplung.00311.2013 PMC3920201 24318116

[32] Cameron MJ Bermejo-Martin JF Danesh A Muller MP Kelvin DJ. Human immunopathogenesis of severe acute respiratory syndrome (SARS). Virus Res (2008) 133(1):13–9. doi: 10.1016/j.virusres.2007.02.014 PMC7114310 17374415

[33] Channappanavar R Perlman S. Pathogenic human coronavirus infections: causes and consequences of cytokine storm and immunopathology. Semin Immunopathol (2017) 39(5):529–39. doi: 10.1007/s00281-017-0629-x PMC7079893 28466096

[34] Luong D Kesharwani P Deshmukh R Mohd Amin MCI Gupta U Greish K. PEGylated PAMAM dendrimers: Enhancing efficacy and mitigating toxicity for effective anticancer drug and gene delivery. Acta Biomat (2016) 43:14–29. doi: 10.1016/j.actbio.2016.07.015 27422195

[35] Tantuoyir MM Rezaei N. Serological tests for COVID-19: Potential opportunities. Cell Biol Int (2021) 45(4):740–8. doi: 10.1002/cbin.11516 PMC7753382 33289157

[36] Wang L He W Yu X Hu D Bao M Liu H. Coronavirus disease 2019 in elderly patients: Characteristics and prognostic factors based on 4-week follow-up. J Infect (2020) 80(6):639–45. doi: 10.1016/j.jinf.2020.03.019 PMC7118526 32240670

[37] Aw D Silva AB Palmer DB. Immunosenescence: emerging challenges for an ageing population. Immunology (2007) 120(4):435–46. doi: 10.1111/j.1365-2567.2007.02555.x PMC2265901 17313487

[38] Li M Yao D Zeng X Kasakovski D Zhang Y Chen S. Age related human T cell subset evolution and senescence. Immun Ageing A (2019) 16:24. doi: 10.1186/s12979-019-0165-8 PMC6739976 31528179

[39] Weng NP Akbar AN Goronzy J. CD28(-) T cells: their role in the age-associated decline of immune function. Trends Immunol (2009) 30(7):306–12. doi: 10.1016/j.it.2009.03.013 PMC2801888 19540809

[40] Panigrahy D Gilligan MM Huang S Gartung A Cortés-Puch I Sime PJ. Inflammation resolution: a dual-pronged approach to averting cytokine storms in COVID-19? Cancer Metastasis Rev (2020) 39(2):337–40. doi: 10.1007/s10555-020-09889-4 PMC7207990 32385712

[41] Zhao Y Qin L Zhang P Li K Liang L Sun J. Longitudinal COVID-19 profiling associates IL-1RA and IL-10 with disease severity and RANTES with mild disease. JCI Insight (2020) 5(13):e139834. doi: 10.1172/jci.insight.139834 32501293 PMC7406242

[42] Soy M Keser G Atagündüz P Tabak F Atagündüz I Kayhan S. Cytokine storm in COVID-19: pathogenesis and overview of anti-inflammatory agents used in treatment. Clin Rheumatol (2020) 39(7):2085–94. doi: 10.1007/s10067-020-05190-5 PMC7260446 32474885

[43] Lu L Zhang H Zhan M Jiang J Yin H Dauphars DJ. Preventing mortality in COVID-19 patients: Which cytokine to target in a raging storm? Front Cell Dev Biol (2020) 8:677. doi: 10.3389/fcell.2020.00677 32766256 PMC7379422

[44] Shi S Qin M Shen B Cai Y Liu T Ysng F. Association of cardiac injury with mortality in hospitalized patients with COVID-19 in wuhan, China. JAMA Cardiol (2020) 5(7):802. doi: 10.1001/jamacardio.2020.0950 32211816 PMC7097841

[45] Gollomp K Kim M Johnston I Hayes V Welsh J Arepally GM. Neutrophil accumulation and NET release contribute to thrombosis in HIT. JCI Insight (2018) 3(18):e99445. doi: 10.1172/jci.insight.99445 30232279 PMC6237233

[46] Pujhari S Paul S Ahluwalia J Rasgon JL. Clotting disorder in severe acute respiratory syndrome coronavirus 2. Rev Med Virol (2021) 31(3):e2177. doi: 10.1002/rmv.2177 33022790 PMC7646030

[47] Varga Z Flammer AJ Steiger P Haberecker M Andermatt R Zinkernagel AS. Endothelial cell infection and endotheliitis in COVID-19. Lancet Lond Engl (2020) 395(10234):1417–8. doi: 10.1016/S0140-6736(20)30937-5 PMC7172722 32325026

[48] Sanders JM Monogue ML Jodlowski TZ Cutrell JB. Pharmacologic treatments for coronavirus disease 2019 (COVID-19): A review. JAMA (2020) 323(18):1824–36. doi: 10.1001/jama.2020.6019 32282022

[49] Stabley DL Harris AW Holbrook J Chubbs NJ Lozo KW Crawford TO. SMN1 and SMN2 copy numbers in cell lines derived from patients with spinal muscular atrophy as measured by array digital PCR. Mol Genet Genomic Med (2015) 3(4):248–57. doi: 10.1002/mgg3.141 PMC4521962 26247043

[50] Pearce L Davidson SM Yellon DM. The cytokine storm of COVID-19: a spotlight on prevention and protection. Expert Opin Ther Targets (2020) 24(8):723–30. doi: 10.1080/14728222.2020.1783243 32594778

[51] Rossello X Yellon DM. The RISK pathway and beyond. Bas Res Cardiol (2018) 113(1):2. doi: 10.1007/s00395-017-0662-x PMC5688212 29143177

[52] Heusch G. Critical issues for the translation of cardioprotection. Circ Res (2017) 120(9):1477–86. doi: 10.1161/CIRCRESAHA.117.310820 28450365

[53] Frangogiannis NG Smith CW Entman ML. The inflammatory response in myocardial infarction. Cardiovasc Res (2002) 53(1):31–47. doi: 10.1016/S0008-6363(01)00434-5 11744011

[54] Hausenloy DJ Yellon DM. Reperfusion injury salvage kinase signalling: taking a RISK for cardioprotection. Heart Fail Rev (2007) 12(3–4):217–34. doi: 10.1007/s10741-007-9026-1 17541822

[55] Davidson SM Ferdinandy P Andreadou I Bøtker HE Heusch G Ibáñez B. Multitarget strategies to reduce myocardial Ischemia/Reperfusion injury: JACC review topic of the week. J Am Coll Cardiol (2019) 73(1):89–99. doi: 10.1016/j.jacc.2018.09.086 30621955

[56] Hausenloy DJ Yellon DM. Ischaemic conditioning and reperfusion injury. Nat Rev Cardiol (2016) 13(4):193–209. doi: 10.1038/nrcardio.2016.5 26843289

[57] Hu B Huang S Yin L. The cytokine storm and COVID-19. J Med Virol (2021) 93(1):250–6. doi: 10.1002/jmv.26232 PMC7361342 32592501

[58] Clinical management of severe acute respiratory infection when novel coronavirus (2019-nCoV) infection is suspected: interim guidance (2020).

[59] Liu D Ahmet A Ward L Krishnamoorthy P Mandelcorn ED Leigh R. A practical guide to the monitoring and management of the complications of systemic corticosteroid therapy. Allergy Asthma Clin Immunol Off J Can Soc Allergy Clin Immunol (2013) 9(1):30. doi: 10.1186/1710-1492-9-30 PMC3765115 23947590

[60] Ye Q Wang B Mao J. The pathogenesis and treatment of the `Cytokine storm’ in COVID-19. J Infect (2020) 80(6):607–13. doi: 10.1016/j.jinf.2020.03.037 PMC7194613 32283152

[61] Menachery VD Schäfer A Burnum-Johnson KE Mitchell HD Eisfeld AJ Walters KB. MERS-CoV and H5N1 influenza virus antagonize antigen presentation by altering the epigenetic landscape. Proc Natl Acad Sci USA (2018) 115(5):E1012–21. doi: 10.1073/pnas.1706928115 PMC5798318 29339515

[62] Ho JC Ooi GC Mok TY Chan JW Hung I Lam B. High-dose pulse versus nonpulse corticosteroid regimens in severe acute respiratory syndrome. Am J Respir Crit Care Med (2003) 168(12):1449–56. doi: 10.1164/rccm.200306-766OC 12947028

[63] Yin-Chun Yam L Chun-Wing Lau A Yuk-Lin Lai F Shung E Chan J Wong V. Corticosteroid treatment of severe acute respiratory syndrome in Hong Kong. J Infect (2007) 54(1):28–39. doi: 10.1016/j.jinf.2006.01.005 16542729 PMC7112522

[64] Stockman LJ Bellamy R Garner P. SARS: systematic review of treatment effects. PloS Med (2006) 3(9):e343. doi: 10.1371/journal.pmed.0030343 16968120 PMC1564166

[65] Guo J Xia H Wang S Yu L Zhang H Chen J. The artificial-liver blood-purification system can effectively improve hypercytokinemia for COVID-19. Front Immunol (2020) 11:586073. doi: 10.3389/fimmu.2020.586073 33424838 PMC7786016

[66] Castellà M. Pilot study on cytokine filtration in COVID-19 ARDS (CytokCOVID19) . Available at: https://clinicaltrials.gov/ct2/show/NCT04361526.

[67] Galán M Rodríguez JS Jiménez JL Relloso M Maly M de la MFJ. Synthesis of new anionic carbosilane dendrimers *via* thiol–ene chemistry and their antiviral behaviour. Org Biomol Chem (2014) 12(20):3222–37. doi: 10.1039/c4ob00162a 24728319

[68] Abdulla ZA Al-Bashir SM Alzoubi H Al-Salih NS Aldamen AA Abdulazeez AZ. The role of immunity in the pathogenesis of SARS-CoV-2 infection and in the protection generated by COVID-19 vaccines in different age groups. Pathogens (2023) 12(2):329. doi: 10.3390/pathogens12020329 36839601 PMC9967364

[69] Sun X Wang T Cai D Hu Z Chen J Liao H. Cytokine storm intervention in the early stages of COVID-19 pneumonia. Cytokine Growth Factor Rev (2020) 53:38–42. doi: 10.1016/j.cytogfr.2020.04.002 32360420 PMC7182527

[70] Kuba K Imai Y Rao S Gao H Guo F Guan B. A crucial role of angiotensin converting enzyme 2 (ACE2) in SARS coronavirus–induced lung injury. Nat Med (2005) 11(8):875–9. doi: 10.1038/nm1267 PMC7095783 16007097

[71] Zhang Y Yu L Tang L Zhu M Jin Y Wang Z. A promising anti-Cytokine-Storm targeted therapy for COVID-19: The artificial-liver blood-purification system. Eng Beijing China. (2021) 7(1):11–3. doi: 10.1016/j.eng.2020.03.006 PMC7118608 32292628

[72] Henderson LA Canna SW Schulert GS Volpi S Lee PY Kernan KF. On the alert for cytokine storm: Immunopathology in COVID-19. Arthritis Rheumatol Hoboken NJ. (2020) 72(7):1059–63. doi: 10.1002/art.41285 PMC7262347 32293098

[73] Chakraborty C Sharma AR Bhattacharya M Sharma G Lee S Agoramoorthy G. COVID-19: Consider IL-6 receptor antagonist for the therapy of cytokine storm syndrome in SARS-CoV-2 infected patients. J Med Virol (2020) 92(11):2260–2. doi: 10.1002/jmv.26078 PMC7283789 32462717

[74] Scharf C Schroeder I Paal M Winkels M Irlbeck M Zoller M. Can the cytokine adsorber CytoSorb® help to mitigate cytokine storm and reduce mortality in critically ill patients? a propensity score matching analysis. Ann Intensive Care (2021) 11(1):115. doi: 10.1186/s13613-021-00905-6 34292421 PMC8295971

[75] Niazi NS Nassar TI Stewart IJ Honore PM Sharma K Chung KK. A review of extracorporeal blood purification techniques for the treatment of critically ill coronavirus disease 2019 patients. Asaio J (2022) 68(10):1219–27. doi: 10.1097/MAT.0000000000001761 PMC9521577 35417433

[76] Träger K Schütz C Fischer G Schröder J Skrabal C Liebold A. Cytokine reduction in the setting of an ARDS-associated inflammatory response with multiple organ failure. Case Rep Crit Care (2016) 2016:9852073. doi: 10.1155/2016/9852073 26885411 PMC4739007

[77] Ankawi G Xie Y Yang B Xie Y Xie P Ronco C. What have we learned about the use of cytosorb adsorption columns? Blood Purif (2019) 48(3):196–202. doi: 10.1159/000500013 31039564

[78] Barbaro RP MacLaren G Boonstra PS Iwashyna TJ Slutsky AS Fan E. Extracorporeal membrane oxygenation support in COVID-19: an international cohort study of the extracorporeal life support organization registry. Lancet Lond Engl (2020) 396(10257):1071–8. doi: 10.1016/S0140-6736(20)32008-0 PMC7518880 32987008

[79] Heymann M Schorer R Putzu A. Mortality and adverse events of hemoadsorption with CytoSorb® in critically ill patients: A systematic review and meta-analysis of randomized controlled trials. Acta Anaesthesiol Scand (2022) 66(9):1037–50. doi: 10.1111/aas.14115 PMC9541789 35788557

[80] Rizvi S Danic M Silver M LaBond V. Cytosorb filter: An adjunct for survival in the COVID-19 patient in cytokine storm? a case report. Heart Lung (2021) 50(1):44–50. doi: 10.1016/j.hrtlng.2020.09.007 33041058 PMC7500900

[81] Morris C Gray L Giovannelli M. Early report: The use of Cytosorb^TM^ haemabsorption column as an adjunct in managing severe sepsis: initial experiences, review and recommendations. J Intensive Care Soc (2015) 16(3):257–64. doi: 10.1177/1751143715574855 PMC5606440 28979423

[82] Al Shareef K Bakouri M. Cytokine blood filtration responses in COVID-19. Blood Purif (2021) 50(2):141–9. doi: 10.1159/000508278 PMC7490911 32464624

[83] Alharthy A Faqihi F Memish ZA Balhamar A Nasim N Shahzad A. Continuous renal replacement therapy with the addition of CytoSorb cartridge in critically ill patients with COVID-19 plus acute kidney injury: A case-series. Artif Organs (2021) 45(5):E101–12. doi: 10.1111/aor.13864 PMC7753655 33190288

[84] Geraci TC Kon ZN Moazami N Chang SH Carillo J Chen S. Hemoadsorption for management of patients on veno-venous ECMO support for severe COVID-19 acute respiratory distress syndrome. J Card Surg (2021) 36(11):4256–64. doi: 10.1111/jocs.15785 PMC8447331 34219277

[85] Song T Hayanga J Durham L Garrison L McCarthy P Barksdale A. CytoSorb therapy in COVID-19 (CTC) patients requiring extracorporeal membrane oxygenation: A multicenter, retrospective registry. Front Med (2021) 8:773461. doi: 10.3389/fmed.2021.773461 PMC8720923 34988092

[86] Sobott F Watt SJ Smith J Edelmann MJ Kramer HB Kessler BM. Comparison of CID versus ETD based MS/MS fragmentation for the analysis of protein ubiquitination. J Am Soc Mass Spectrom (2009) 20(9):1652–9. doi: 10.1016/j.jasms.2009.04.023 19523847

[87] Ricci Z Romagnoli S Reis T Bellomo R Ronco C. Hemoperfusion in the intensive care unit. Intensive Care Med (2022) 48(10):1397–408. doi: 10.1007/s00134-022-06810-1 PMC9389493 35984473

[88] Villa G Zaragoza JJ Sharma A Neri M De Gaudio AR Ronco C. Cytokine removal with high cut-off membrane: review of literature. Blood Purif (2014) 38(3–4):167–73. doi: 10.1159/000369155 25471681

[89] Ruotolo BT Gillig KJ Stone EG Russell DH Fuhrer K Gonin M. Analysis of protein mixtures by matrix-assisted laser desorption ionization-ion mobility-orthogonal-time-of-flight mass spectrometry. Int J Mass Spectrom (2002) 219(1):253–67. doi: 10.1016/S1387-3806(01)00583-8

[90] Ronco C Bagshaw SM Bellomo R Clark WR Husain-Syed F Kellum JA. Extracorporeal blood purification and organ support in the critically ill patient during COVID-19 pandemic: Expert review and recommendation. Blood Purif (2021) 50(1):17–27. doi: 10.1159/000508125 32454500 PMC7270067

[91] Stasi A Franzin R Divella C Sallustio F Curci C Picerno A. PMMA-based continuous hemofiltration modulated complement activation and renal dysfunction in LPS-induced acute kidney injury. Front Immunol (2021) 12:605212. doi: 10.3389/fimmu.2021.605212 33868226 PMC8047323

[92] Asgharpour M Mehdinezhad H Bayani M Zavareh MSH Hamidi SH Akbari R. Effectiveness of extracorporeal blood purification (hemoadsorption) in patients with severe coronavirus disease 2019 (COVID-19). BMC Nephrol (2020) 21(1):356. doi: 10.1186/s12882-020-02020-3 32819292 PMC7439633

[93] Kielstein JT Borchina DN Fühner T Hwang S Mattoon D Ball AJ. Hemofiltration with the seraph® 100 microbind® affinity filter decreases SARS-CoV-2 nucleocapsid protein in critically ill COVID-19 patients. Crit Care Lond Engl (2021) 25(1):190. doi: 10.1186/s13054-021-03597-3 PMC8169409 34074339

[94] Chen G Zhou Y Ma J Xia P Qin Y Li X. Is there a role for blood purification therapies targeting cytokine storm syndrome in critically severe COVID-19 patients? Ren Fail (2020) 42(1):483–8. doi: 10.1080/0886022X.2020.1764369 PMC7946020 32438839

[95] Lu W Kelley W Fang DC Joshi S Kim Y Paroder M. The use of therapeutic plasma exchange as adjunctive therapy in the treatment of coronavirus disease 2019: A critical appraisal of the current evidence. J Clin Apheresis (2021) 36(3):483–91. doi: 10.1002/jca.21883 PMC8014837 33578448

